# Blood Transfusion

**Published:** 1951-01

**Authors:** 


**Blood Transfusion**

"It is a mistake to suppose, as many of us do, that specialism in medicine or surgery is a modern innovation. . . . Great contributions to recent progress have mostly come from those who have had large opportunities for practical experience in relatively restricted fields." These sentences, piously quoted from a Presidential Address of nearly forty years ago, remind us that even then human knowledge had become so extensive that no one man could be a master of the whole art of healing, or even of one of the major branches.*

The story of blood transfusion well illustrates the advances achieved by, and the necessity for, specialization. Formerly too dangerous to contemplate, the procedure became practical and valuable with the discovery of the well-known but variously named "four groups". "M and N" added a little to the complexity, and the numerous Rh factors very much. But what do we make of "Group B Rh-positive (CDe/CDe), MN, p, $Le^a$-positive, Kell-positive, Cellano-positive (presumably Kk)"?

Our esteemed elder brother, the *British Medical Journal*, has recently† directed attention to yet another hazard. During the last twelve years the use of blood and plasma transfusion has multiplied fifty times: and viral hepatitis is already a real menace. The virus is not very rare; there is no known method of inactivating it or even of detecting it before administration: apparently, therefore, anyone receiving a carefully "typed and matched" transfusion runs a one in ten risk of contracting a disease which is often crippling and not seldom fatal. It may well prove necessary to reorganize the whole system of "blood-banks", plasma distribution and so forth. But clearly we are back once again at the point where transfusion is to be risked only where it is essential to save life.

* "Each physician applies himself to one disease only, and not more. Some are physicians for the eyes, others for the head . . . . others for internal disorders." Herodotus II, 84.

† 11.11.50, p. 1103.

